# Hormonal contraception increases the risk of psychotropic drug use in adolescent girls but not in adults: A pharmacoepidemiological study on 800 000 Swedish women

**DOI:** 10.1371/journal.pone.0194773

**Published:** 2018-03-22

**Authors:** Sofia Zettermark, Raquel Perez Vicente, Juan Merlo

**Affiliations:** Unit of Social Epidemiology, Department of Clinical Sciences, Lund University, Clinical Research Center, Malmö, Sweden; Karolinska Institutet, SWEDEN

## Abstract

The burden of depression and anxiety disorders is greater in women, and female sex hormones have been shown to affect mood. Psychological side effects of hormonal contraception (HC) are also a common complaint in the clinic, but few previous studies have investigated this subject. We therefore wanted to investigate whether use of HC was associated with adverse psychological health outcomes, and whether this association was modified by age. All women aged 12–30 years on 31 December 2010, residing in Sweden for at least four years and with no previous psychiatric morbidity (n = 815 662), were included. We followed the women from their first HC use (or 31 December 2010, if they were non-users) at baseline, until a prescription fill of psychotropic drugs or the end of the one-year follow-up. We performed age-stratified logistic regression models and estimated odds ratios (OR) to measure the association between different HC methods and psychotropic drug use, as well as the area under the receiver operating curve to estimate discriminatory accuracy of HC in relation to psychotropic drugs. Overall, we found an association between HC and psychotropic drugs (adjusted OR 1.34, 95% confidence interval [CI] 1.30–1.37). In the age-stratified analysis, the strongest association was found in adolescent girls (adjusted OR 3.46, 95% CI 3.04–4.94 for age 12 to 14 years), while it was non-existent for adult women. We conclude that hormonal contraception is associated with psychotropic drug use among adolescent girls, suggesting an adverse effect of HC on psychological health in this population.

## Introduction

In clinical practice and among the general public, psychological side effects are a well-known factor for dissatisfaction with hormonal contraception (HC) [1]. Discontinuation rates of HC are high, especially among adolescents, and mood complaints are one of the most frequently stated reasons for discontinuation [2, 3]. Depression and anxiety disorders are twice as prevalent in women as in men; a difference that is yet to be fully understood [4–6] and rates of psychiatric diagnosis and visits to mental health professionals are increasing in young Swedish women [7]. The gender disparity in psychological health does not exist in children but starts at the onset of puberty, rendering female sex hormones one plausible explanation [8]. Several mechanisms have been suggested to explain the mood-altering effect of these hormones. Estrogen is a regulator of serotonin levels, a known factor in depression [9, 10]. Progesterone most likely influences mood through the action of its neuroactive metabolites, to which not all women respond in the same way despite similar levels [11].

The scientific literature on psychological side effects of HC is relatively scant and inconclusive. Few randomized controlled trials have been performed, but most existing studies show a protective association between the most prevalent HC, combined oral contraception (COC), and anxiety, mood disturbances and depressive symptoms [12, 13]. The field is, however, disputed, and recent studies have also found adverse mental health effects [14, 15]. A new Danish register-based cohort study found an association between HC and subsequent use of antidepressants and a hospital diagnosis of depression, which was higher in adolescents [16]. This could be due to a *healthy survivor effect*, or more correctly, *a selective discontinuation bias*, where only women content with their treatment continue, creating a false protective association in older women.

Against the above background we aimed to investigate a possible adverse effect of HC on psychological health, using a nationwide cohort of 815 662 women during 2010–2011. Our main hypothesis was that this association would be stronger in adolescents since, being new users of HC, this population should be less affected by a *selective discontinuation bias*.

## Methods

### Population

We designed a nationwide database by record linkage of several Swedish registers, using the official unique personal identification number assigned to every individual residing in Sweden [17]. The database was constructed by Statistics Sweden and the Swedish National Board of Health and Welfare, and it was delivered to us after replacing the personal identification number by an arbitrary one to ensure anonymity of subjects. Based on the Total Population Register, we defined an initial cohort containing all 1 094 069 women aged 12–30 years residing in Sweden on 31 December 2010 [18]. We obtained information on medication use from the Swedish Prescribed Drug Register (SPDR), which contains individual level data on all dispensed drug prescriptions at Swedish pharmacies (excluding dispensation at hospitals and nursing homes) for the whole population in Sweden since 2006 [19].

We assigned a baseline date to every woman, defined by the first dispensed prescription of an HC drug (Anatomical Therapeutic Chemical (ATC) codes G02BA, G02BB, G03AA, G03AB, G03AC) between 1 January 2010 and 31 December 2011. The baseline date was set to 31 December 2010 if the woman did not have any HC prescription during these two years. Each woman, user and non-user, thereby got an individual baseline date based on her first prescription fill or the assigned date of 31 December 2010. From the individual baseline date, a one-year follow-up and a previous period of four years were recorded, meaning all women were followed for exactly one year. With this definition, we can furthermore be sure that the non-users of HC were true non-users during four years before, and one year after their baseline date of 31 December 2010.

After calculating the individual baseline date, we excluded 99 052 women who were not residing in Sweden for four years prior to, and one year after their own baseline date, since information on diagnosis and medication were lacking in those cases. Women with pre-existing psychiatric disorders were excluded by using information on diagnoses from hospital discharges and outpatient visits recorded at the Swedish Patient Register. Through the International Classification of Diseases, 10^th^ version (ICD-10), women with a psychiatric diagnosis (ICD-10 codes F00–F99) in the previous four years were excluded. Since diagnosis from primary care visits are not available in Swedish registers, we also used information from the SPDR, which includes prescriptions from primary care as well as specialised care to identify women with less severe mental health issues. Women having a prescription fill of a psychotropic drugs (ATC N05 or N06) in the previous four years were therefore also excluded (n = 156 302). The period of four years was determined because of restrictions in the available information on medications. Finally, we excluded 23 053 women who had a diagnosis of child delivery (ICD-10 O80-84) at a hospital during follow-up. The final cohort consisted of 815 662 women.

### Assessment of variables

#### Hormonal contraceptive use

We defined *non-users* of HC as those who did not fill a prescription for HC between 1 January 2010 and 31 December 2011, and took this category as reference in all comparisons with *users of HC*. Users of HC were identified according to the first HC prescription fill during baseline.

Since HC can only be acquired via a prescription from a physician or midwife in Sweden all users were included. The exception is emergency contraceptives that are mainly dispensed over the counter and are therefore not registered in the SPDR. A small number, 288 prescription fills of emergency contraceptives, were recorded in the SPDR, but were excluded from the analysis. HC prescriptions in Sweden are filled every three months or annually.

#### Psychotropic drug use

The outcome of our study was use of psychotropic medication defined as filling at least one prescription with anxiolytics, hypnotics and sedatives or anti-depressants (ATC N05B, N05C or N06A) during the one-year follow-up, from the first dispensation of HC or 31 December 2010, if the individual did not use HC at baseline. Use of psychotropic medication can be considered as a proxy for impaired psychological health [20].

#### Socioeconomic position

We obtained individual and family level data on socioeconomic factors as of 31 December 2010 from Statistics Sweden’s *Longitudinal integration database for health insurance and labour market studies* and operationalized SEP by means of education and income [21]. Education was categorized according to the number of years into low (less than 14 years) and high (14 years or more) level of educational achievement, with a category for missing values. We used the highest educational level achieved by any member of the woman’s current household, since many individuals in our cohort were children. Individualized disposable family income was calculated by dividing the total disposable income of the family by the number of family members, taking into account the different consumption weights of adults and children determined by Statistics Sweden. Thereafter, we created three categories (i.e., low, medium, and high) of income using the total Swedish population aged 18–80 years. We considered the high income category as the reference in the comparisons.

#### Other variables

We categorized age into the following groups; 12–14, 15–17, 18–20, 21–25 and 26–30 years, as well as into adolescents (12–19 years) and adult women (20–30 years) and used these age categorizations to perform different stratified analyses. We created dichotomous variables to adjust for the presence or absence of any previous diagnosis related to thromboembolism (ICD-10 I26, I80 and I82), epilepsy or migraine (ICD-10 G40-47) and menstrual disturbances including abnormal bleeding, premenstrual disorders and endometriosis (ICD-10 N80 and N91-94) in the previous four years. Information on diagnosis were not available from primary health care clinics. Contraindication of certain HCs for women with these diagnoses, and presence of menstrual disturbances, could affect both HC prescription and mood and therefore confound the results. Thereafter, we adopted a more exhaustive approach and adjusted for the presence or absence of any hospitalization or contact with an outpatient hospital clinic in the past four years. In the case that a potential association between HC use and use of psychotropic medication were mediated by an increased contact with health care services, our results would underestimate this association. Since the use of psychotropic drugs rises with age, we also adjusted for age as a continuous variable within each age stratum.

#### Statistical analysis

Since the follow-up was short and complete, we applied logistic regression to estimate odds ratios (OR) and 95% confidence interval (CI) of psychotropic drug use in relation to the different HC categorizations, with non-users of HC as the reference group. We performed simple and multiple age-stratified logistic regression models.

In the multiple regression models, 2545 cases were dropped due to missing information on family education level.

We obtained the predicted probabilities from the models to calculate the area under the operating receiver operator characteristics curve (AUC) [22]. The AUC measures the ability of the model to correctly classify those using or not using psychotropic medication, based on knowledge of HC use. The AUC assumes a value between 0.5 and 1, where 1 represents perfect discrimination and 0.5 corresponds with a predictive value equal to that obtained by flipping an unbiased coin.

We performed all analyses using SPSS 22.0 (Statistical Package for Social Sciences, SPSS Inc., Chicago, IL, USA) software.

### Ethical approval

The database was approved by the Regional Ethical Review Board in Lund, Sweden, the Data Safety Board at Statistics Sweden and the National Board of Health and Welfare (Dnr: 2014/856, 2015/341).

## Results

The study population consisted of 815 662 women aged 12–30 years (mean age 20.44 years; standard deviation (SD) 5.3) on 31 December 2010, residing in Sweden since at least four years and having no previous psychiatric morbidity. Overall, 49.5% (n = 411 559) of the women were users of HC. Psychotropic drugs were dispensed to 3.1% (n = 24 973) of the women during the follow-up. Among HC users the incidence of psychotropic drug use was 3.7%, while this figure was 2.5% for non-users. For detailed information on prevalence and types of HC included, see S1 Table.

### Hormonal contraceptive users

The age-stratified baseline characteristics of the population are presented in Table 1. The mean age for non-users of HC was 19.3 years (SD 5.8), and users were somewhat older, with a mean age of 21.6 years (SD 4.4). We did not find any major differences between the groups at baseline. Health care utilization was higher among HC users (21.7% vs. 17.9% had been hospitalized), and so was the frequency of menstrual disorders (4.1% vs. 2.8%).

**Table 1:** Prevalence of different hormonal contraceptive methods in our cohort. Use of hormonal contraception, socioeconomic characteristics, contact with health care, and previous diagnoses at baseline (2010–2011) by age groups and use of hormonal contraceptives in our cohort of 815 662 Swedish women. Values are percentages, unless otherwise indicated.

Age (years)	12–19		20–30		All	
Number of women	380 818		434 844		815 662	
	Non-user	User	Non-user	User	Non-user	User
Hormonal contraceptive	-	40.1	-	59.5	-	49.5
*Income*						
Low	52.5	46.3	39.0	30.3	46.6	36.2
Middle	33.4	37.3	34.9	38.0	34.1	37.8
High	14.1	16.4	26.1	26.1	19.3	26.0
*Education*						
Low	48.2	57.2	46.4	46.4	47.1	50.8
High	51.5	42.7	53.0	53.0	52.2	49.0
Missing data	0.2	0.1	0.7	0.4	0.4	0.2
*Health care*						
Hospitalizations	8.1	12.1	30.9	27.4	17.9	21.7
Outpatient care	50.5	59.8	62.4	65.4	55.7	63.6
Diagnoses						
Thrombosis	0.0	0.1	0.2	0.2	0.1	0.1
Epilepsy or migraine	1.3	1.4	1.3	1.2	1.3	1.2
Menstrual disturbances	1.2	3.7	4.9	4.3	2.8	4.1

Patterns of HC use varied with age. In the youngest age stratum, 7.77% were HC users, while as many as 48.8% of the girls aged 15–17 years and 68.2% of the women aged 18–20 years used HC during 2010–2011.

### Hormonal contraception and psychotropic drug use

**Fig 1:**
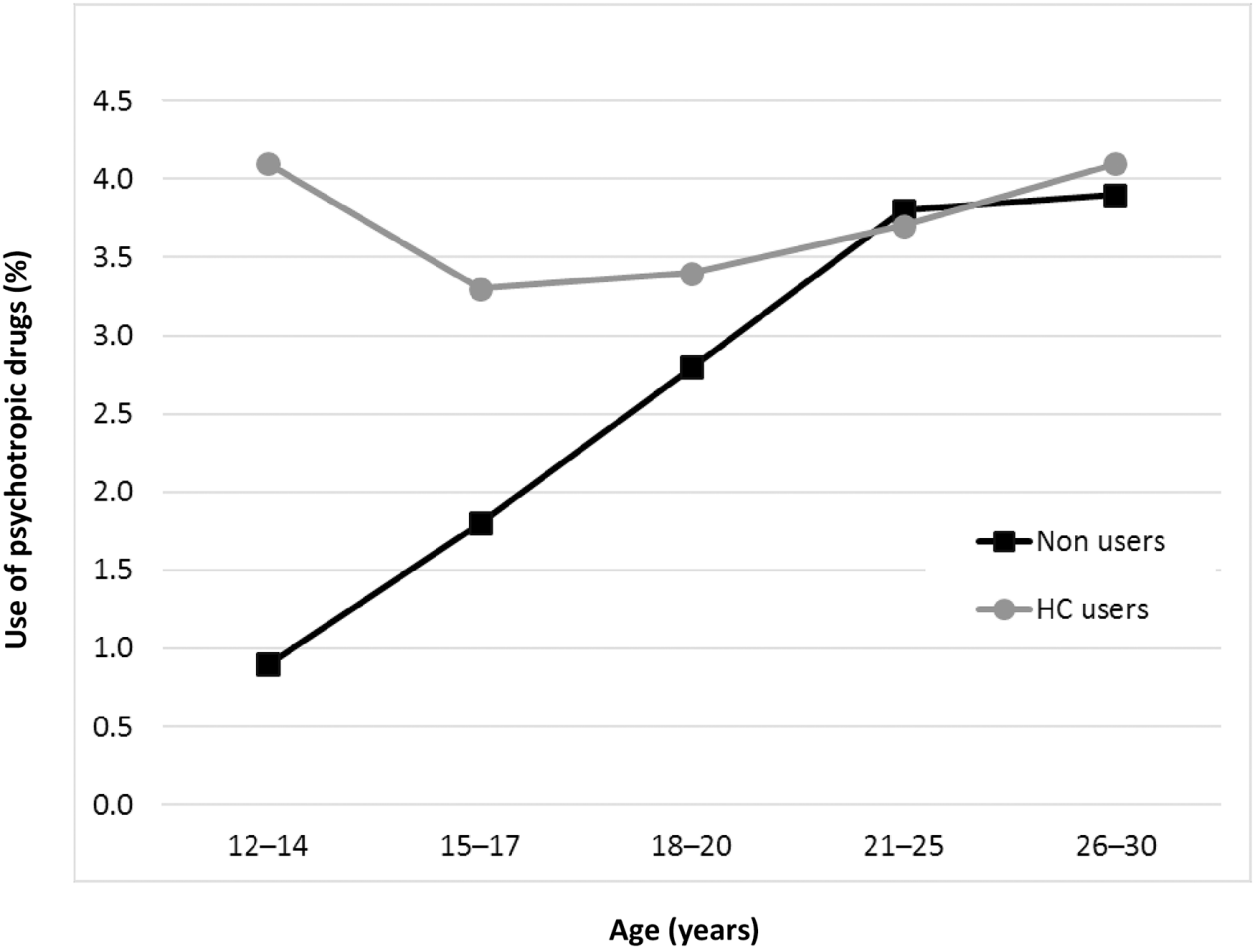
Psychotropic drug use in users and non-users of hormonal contraception. Percentage (i.e. absolute risk) of first-time use of psychotropic drugs during a one-year follow-up from baseline (2010–2011) by age, in users (gray line with circles) and non-users (black line with squares), of hormonal contraceptives in 815 662 Swedish women.

In users of HC the absolute risk of using psychotropics was around 4% for all age groups (Fig 1). In non-users of HC, however, this risk was very low in young adolescents and increased with age, to reach the level of HC users at the age of 21 or older.

**Fig 2:**
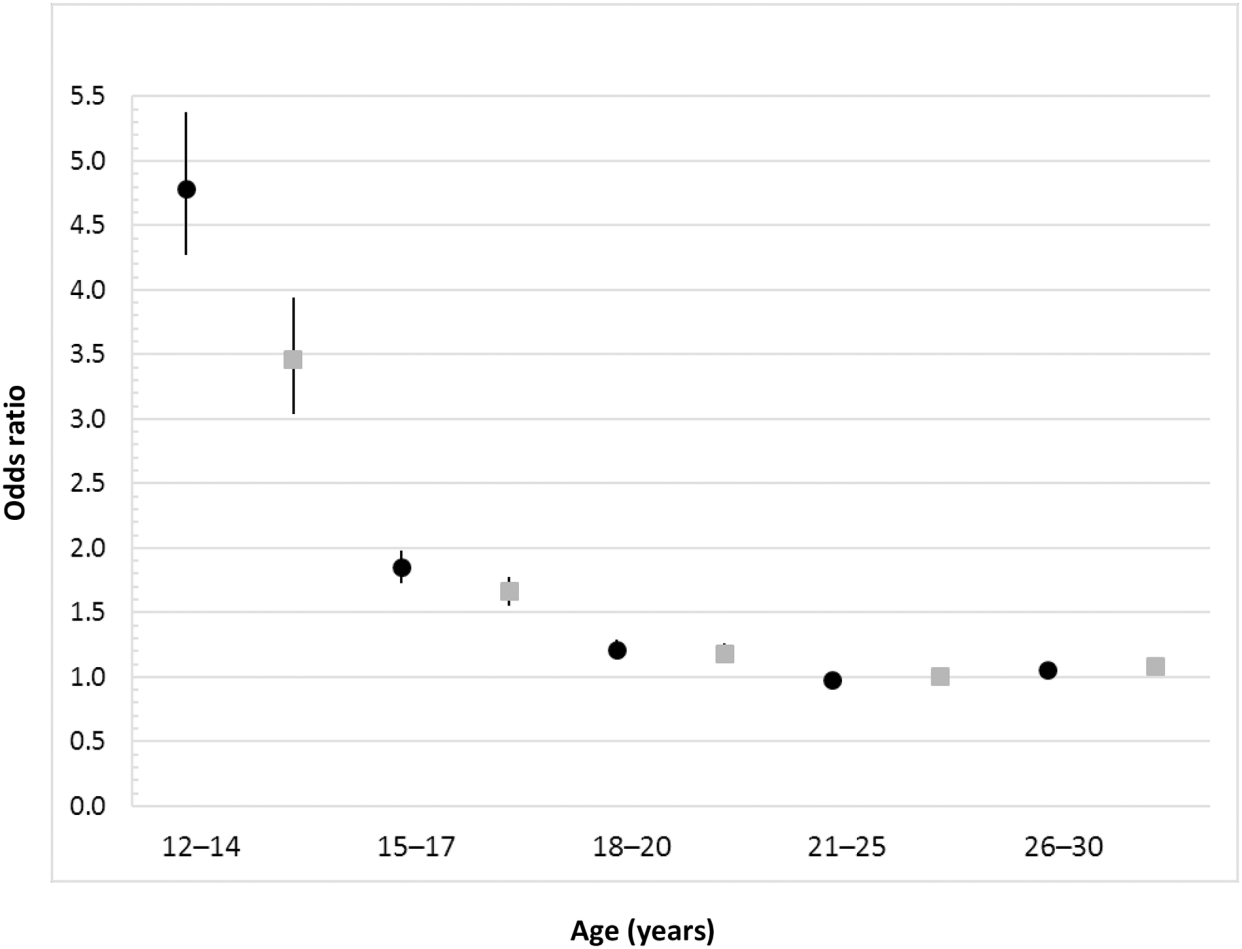
Age-stratified association between use of hormonal contraception and a first-time use of psychotropic drugs. Age-stratified odds ratios with 95% confidence intervals (black lines) for the association between use of hormonal contraceptives and use of psychotropic drugs within a one-year follow-up after baseline (2010–2011) in 815 662 Swedish women. Crude (black circles) and adjusted (gray squares) values. Adjustments were made for age, family income, highest educational level in family, previous hospitalizations, outpatient hospital visits, and having a diagnosis of thromboembolism, epilepsy or migraine, or menstrual disturbances including endometriosis.

Overall, compared to non-users, users of HCs had an adjusted OR of a first-time use of psychotropic drugs of 1.34 (95% CI: 1.30–1.37). Adjustments were made for age, family income, educational level, health care utilisation, and having a diagnosis of thromboembolism, epilepsy or migraine, or menstrual disturbances. However, the age-stratified analysis (Fig 2) revealed a strong association in adolescent girls, which decreased with age to disappear after adolescence. We observed this pattern for all groups of HC. The highest ORs were found in the youngest age group of 12- to 14-year-olds, with an adjusted OR of 3.46 (95% CI: 3.04 to 3.94).

In the analysis differentiating between oral and non-oral forms of combined and progesterone-only methods, we observed a pattern where use of HC in young adolescents displayed strong associations with psychotropic drug use (Table 2). The strongest association was found among 12–14 year olds using a non-oral progesterone-only method such as a skin patch or intravaginal ring, with an of OR 4.47 (95% CI: 2.08–8.78). The non-oral methods generally had higher ORs than the oral methods, as did the progesterone-only methods compared to combined methods regardless of administrative route. For example, users of the vaginal ring/patch in the age group 15–17 years had an adjusted OR of 2.27 (95% CI: 1.85–2.79) for a first use of psychotropic drugs compared to non-users, while COC users had an OR of 1.52 (95% CI: 1.41–1.64) in the same age group.

**Table 2:** Association between different oral and non-oral hormonal contraceptive methods and use of psychotropic drugs within one-year follow-up. Adjusted age-stratified odds ratios (OR) with 95% confidence intervals (CI) for the association between oral and non-oral hormonal contraception (HC) methods and use of psychotropic drugs within one-year follow-up after baseline (2010–2011) in 815 662 Swedish women. Non-users used as reference within each age group. The analyses distinguish between combined and progesterone-only methods within the oral and non-oral forms of contraceptives. Adjustments were made for age, family income, highest educational level in family, previous hospitalizations, outpatient hospital visits, and having a diagnosis of thromboembolism, epilepsy or migraine, or menstrual disturbances including endometriosis.

Age (years)	Oral HC			Non-oral HC		
		OR (95% CI)	AUC (95% CI)		OR (95% CI)	AUC (95% CI)
12–14	Non-users	1.00 (ref)		Non-users	1.00 (ref)	
	COC	3.3 (2.85–3.81)	0.70 (0.68–0.71)	Patch/ring^a^	4.27 (2.08–8.78)	0.65 (0.63–0.67)
	POP	3.9 (3.14–4.84)		IUD/inj/impl.^b^	3.37 (2.10–5.40)	
15–17	Non-users	1.00 (ref)		Non-users	1.00 (ref)	
	COC	1.52 (1.41–1.64)	0.63 (0.62–0.64)	Patch/ring	2.27 (1.85–2.79)	0.64 (0.63–0.66)
	POP	1.83 (1.65–2.03)		IUD/inj/impl.	2.48 (2.10–2.94)	
18–20	Non-users	1.00 (ref)		Non-users	1.00 (ref)	
	COC	1.08 (1.01–1.16)	0.61 (0.60–0.62)	Patch/ring	1.42 (1.24–1.62)	0.62 (0.61–0.64)
	POP	1.29 (1.18–1.41)		IUD/inj/impl.	1.58 (1.39–1.80)	
21–25	Non-users	1.00 (ref)		Non-users	1.00 (ref)	
	COC	0.94 (0.89–1.00)	0.60 (0.59–0.60)	Patch/ring	1.24 (1.13–1.36)	0.60 (0.59–0.61)
	POP	1.00 (0.93–1.07)		IUD/inj/impl.	1.12 (1.00–1.24)	
26–30	Non-users	1.00 (ref)		Non-users	1.00 (ref)	
	COC	1.10 (1.03–1.17)	0.59 (0.58–0.59)	Patch/ring	1.30 (1.16–1.46)	0.60 (0.59–0.61)
	POP	0.97 (0.91–1.04)		IUD/inj/impl.	1.19 (1.07–1.32)	
All	Non-users	1.00 (ref)		Non-users	1.00 (ref)	
	COC	1.29 (1.26–1.33)	0.65 (0.65–0.65)	Patch/ring	1.57 (1.45–1.67)	0.69 (0.68–0.69)
	POP	1.28 (1.24–1.33)		IUD/inj/impl.	1.46 (1.38–1.55)	

HC; Hormonal contraceptive, COC; combined oral contraceptives, POP; progesterone-only pills, Patch; skin patch (Evra), Ring; intravaginal ring (NuvaRing), IUD; Intrauterine device, Inj.; injection (Depo-Provera), Impl.; implant (Implanon, Jadelle)*

^a^Non-oral combined methods.

^b^Non-oral progesterone-only methods

The analysis of separate HC types showed distinct differences between adolescents (12–19 years) and adult women (20–30 years) (S2 Table). While the majority of HC substances where positively associated with a first-time use of psychotropic drugs in adolescents, we observed few conclusive associations in adult users. Non-oral HC forms did however display stronger associations in both adults and adolescent users.

Use of the depot medroxyprogesterone acetate (DMPA) injection (ATC G03AC06), a high dose progesterone-only method, had the strongest association with subsequent use of psychotropic drugs in adult women, with an adjusted OR of 1.56 (95% CI: 1.34–1.82). For adolescents, the levonorgestrel containing IUD had the strongest association with use of psychotropic drugs, with an adjusted OR of 2.90 (95% CI: 2.22–3.79), followed by DMPA (OR: 2.37; 95% CI: 1.46–3.84).

We also performed a sensitivity analysis excluding women diagnosed with a number of illnesses (i.e., thromboembolism, endocrine disorders, migraine or epilepsy, disorders of the female genital tract, and malignant diseases) that could influence both use of HC and of psychotropic drugs, and the results were very similar (S3 Table).

### Area under the receiver operating curve

Finally, calculations of the AUC showed that the ability to categorize users of psychotropic drugs based on information on HC use was very low. In women over 18 years, the AUCs ranged between 0.54 and 0.60 for unadjusted and adjusted values, respectively (S2 Table). For adolescents the AUC was somewhat higher with values between 0.62 and 0.68 in the youngest age stratum. We also ran a model with all the explanatory variables except HC, and then added use of HC in a consecutive model, to be able to assess how much knowledge of HC use added to the AUC (S4 Table). This analysis showed that HC added nothing to the AUC in women over 18 years of age. In adolescent girls, knowledge of HC added between 0.02 and 0.04 units to the AUC of the model containing only individual predictors.

## Discussion

Our findings show strong associations between the majority of hormonal contraceptives and subsequent use of psychotropic drugs in adolescent girls without previous psychiatric morbidity. We found the highest odds ratios in 12- to 14- year old girls using non-oral progesterone-only methods. The association was persistent and conclusively high for most HC formulas in adolescents, but it weakened or became absent for women over 19 years.

### A possible selective discontinuation bias

Our results could be explained by a *selective discontinuation bias*. As an expression of a heterogeneous response to HC, the effect of HC on psychological health may vary due to, for instance, personality traits, disorders such as premenstrual dysphoric disorder (PMDD) or dysmenorrhea, or different sensitivity for neuroactive metabolites of progesterone [13, 23–26]. Some women might therefore experience a negative influence of HC on psychological health and discontinue treatment, while those without symptoms continued on HC into adulthood, which would explain the lower association with psychotropic drugs in older women. This could explain why previous observational studies focused on adult women have found that HC has a protective effect on adverse psychological symptoms [27–29], while several other studies have found negative influences on mood, especially from progesterone only methods [14, 30–32]. A recent Swedish randomized controlled trial investigating the effect of a second generation COC also found an adverse effect on sexuality [15].

Our results validate the previously reported cross-sectional association between HC and psychotropic drug use as the association remains when temporality between exposure and outcome is taken into account [31, 33]. Our results are furthermore in line with a recent Danish study applying a similar design as the one used by us, which found a high risk of antidepressant consumption in teenagers using HC in general and progesterone-only HCs in particular [16]. Our comparable results demonstrate that these associations are not country specific, and therefore do not depend on, for example, different prescribing cultures. Even if Sweden and Denmark are both Scandinavian countries, their respective health care systems are not identical. Furthermore, our study complements the Danish investigation by including younger girls, among which the association between HC and psychotropic drugs had not previously been investigated.

### An age-dependent heterogeneity in response to HC

A possible mechanism that could explain our results, apart from a *selective discontinuation bias*, is that adolescents are more sensitive to exogenous hormones. One study of 14-to 17-year-old girls found that they exhibited adverse mood effects while on DMPA, compared to non-users as well as oral contraceptive users, and concluded that adolescents react differently than adults to HC [34]. DMPA displayed one of the strongest associations with psychotropic drugs in our study. Another randomized study performed on adolescents with dysmenorrhea found no differences between users of a second generation COC and placebo treatment regarding depressive symptoms [35]. An interesting finding in relation to our results, if still only confirmed in an animal model, is that female mice react differently to allopregnanolone during puberty. The usual sedative effect instead becomes increased anxiety, due to an upregulation of a certain GABA receptor at the onset of puberty [36].

Another route of explanation is found within the social realities of adolescents using HC. It has been concluded that an early sexual debut is associated with augmented risks of destructive behaviour and poor mental health [37]. Since use of HC is a reasonable proxy for an active sex life, the effect of beginning treatment at an early age might instead refer to the effect of early sexual debut. Another aspect of this issue is that HC is used not only for pregnancy prevention, but also for somatic disorders or symptoms such as dysmenorrhea and PMDD, indications that could be more common in adolescents and adversely affect mood in itself. In one Australian cohort study on women in their 20s, the association between HC and depressive symptoms disappeared when controlling for confounding in the form of non-contraceptive use of HC [38].

Our subanalysis of oral and non-oral HC shows a consistent pattern of non-oral forms being more strongly associated with psychotropic drug use (except in the youngest age stratum, where the cases are few and, therefore, the confidence intervals are wide), despite the fact that these methods often contain lower levels of hormones. A plausible assumption is that girls and women in need of a method less dependent on remembering a pill every day might belong to a more vulnerable population. Indeed, adolescents with depression have been found to be more likely to select an IUD as their contraceptive method [39]. Users of levonorgestrel IUDs also report higher rates of anxiety and non-clinical depression, but clinical depression is not more prevalent among users [40]. This can be contrasted against a systematic review that concluded that users of the vaginal ring reported less depression, irritability, and emotional liability than COC users [41]. However, in our study the association with psychotropic drugs was still high for progesterone-only pills for adolescents, which indicates that the effect cannot be exclusively attributed to administrative route.

### Strengths

A strength in our study is the inclusion of all women aged 12–30 years living in Sweden 2006–2013, where the individual woman is followed for one year after her first dispensed HC prescription. This design makes it possible to define the temporality of the exposure and outcome, although causation is difficult to determine by observational studies. Inclusion of girls and women in an age-stratified analysis also makes it possible to derive age-specific patterns of use and associations. Moreover, we excluded women with a previous psychiatric diagnosis made at a hospital visit or use of psychotropic drugs, to be able to focus on the possible effect of HC without the confounding of previous psychiatric illness. Including sedatives and hypnotics in the outcome could also capture a broader spectrum of adverse psychological effects than just focusing on antidepressants, as with previous studies [16, 31, 33]. Finally, a benefit of register data is that it is free from recall bias. Misclassification of information can still occur, but since the SPDR gathers its data directly from the pharmacies’ computer systems, this is a minor problem.

### Limitations

An apparent limitation for a study with the objective of measuring effects on mental health is the use of a crude proxy such as psychotropic medication. A study investigating indications of antidepressants in general practice in the Netherlands found that they were most commonly prescribed for depression, anxiety/panic disorder, and sleeping disorders, but other rare indications were neuropathic pain, headache, and enuresis, meaning that such prescription could not comprise a direct proxy for mental health [20]. Also, with prescription fill data, it is not possible to determine whether the individual took the medicine, although it allows for analysis of large population datasets. Another aspect to consider is that not all women with adverse mental health effects of HC would have symptoms severe enough to get a prescription for a psychotropic drug, leading to many missed cases. This limitation would therefore suggest that our results are an underestimation of the problem at hand. Primary care diagnosis of psychiatric disorders was not available since it is not registered in national registers, which limits the scope of and conclusions possible to draw from our analysis. It is possible that in some women psychological distress which did not warrant psychotropic drugs or a psychiatric diagnosis made at a hospital visit could have predated the prescription fill of HC, which we cannot tell with available data. An additional limitation is the grouping of HC in the age-stratified analysis, which does not allow for investigation of specific formulas, but which, on the other hand, could disclose larger patterns of hormonal content and psychological health and allow for analysis in the adolescent population where the use is low.

The strong association found in adolescent girls could also be a result of an unadjusted confounding, for example, HC as a marker for living conditions, social circumstances that increase the risk of depressive or neurotic symptoms or non contraceptive uses for HC [37]. In our study, the associations held firm when controlling for menstrual disturbances, which could be read as proxy for non-contraceptive uses of HC, and a sensitivity analysis excluding women with these diagnoses did not change the results. However, some women with these diagnoses, as with psychiatric diagnosis, would have gotten them through the primary care system, from where data were not available to us. Due to this fact, as well as the general limitation of register-based data, the variables included in our adjusted analysis were neither optimal nor exhaustive.

Finally, even with adjustments the observational nature of our study only allows for measurements of associations and cannot determine causation. An important aspect of epidemiological research is the ability to accurately interpret and weigh one’s findings and appreciate that high odds ratios for a certain risk factor do not necessarily equate to clinical importance [42]. One method for acquiring this information is to use measures of discriminatory accuracy like the AUC, which reveal with what accuracy individuals can be categorized into groups, depending on certain factors [22]. In our analysis, the discriminatory accuracy for the unadjusted analysis was low, and adding HC to the other explanatory variables increased the AUC only marginally, meaning that we need to seek additional explanations aside from use of HC, to understand the high use of psychotropic drugs and poor psychological health in young girls.

## Conclusion

We find an association between hormonal contraception and subsequent use of psychotropic drugs for women of reproductive age. The association is large for young adolescents, and insignificant in adult women, pinpointing the need to bear population heterogeneity in mind and identifying adolescent girls using hormonal contraception as a vulnerable population. Our also study adds to a growing body of evidence that hormonal contraception could adversely affect psychological health in certain girls and women, and warrants further investigation of the influence of different hormonal contraception on psychological health, particularly in young women.

## Supporting information

S1 Table Prevalence of different hormonal contraceptive methods in our cohort. Prevalence of different hormonal contraceptive methods in 2010–2011 by contraceptive type in our cohort of 815 662 Swedish women aged 12–39. All combined HC contain estrogen in addition to progesterone, except for G03AA14 (Zoely), which contains estradiol.
(DOCX)

S2 Table Association between use of different hormonal contraceptives and a first time use of psychotropic drugs. Odds ratios (OR) with 95% confidence intervals (CI) and area under the curve (AUC), stratified on adolescents and adults, for the association between use of different hormonal contraceptives and a first time use of psychotropic drugs within a one-year follow-up after baseline (2010–2011) in 815 662 Swedish women.
(DOCX)

S3 Table Sensitivity analysis. **Sensitivity analysis excluding women with certain diagnosis**. Odds ratios (OR) with 95% confidence intervals (CI) for the use of hormonal contraceptives (HC) (ATC G02BA, G02BB, G03AA, G03AB, G03AC) and use of psychotropic drugs (ATC N05B, N05C, N06A) within a one-year follow-up after baseline (2010–2011) in 815 662 Swedish women. A sensitivity analysis excluding women with different illnesses (delivery: ICD-10 O80-84, pregnancy and delivery complications: ICD-10 O00-O99, menstrual disturbances; ICD-10: N80 and/or N91- 94, endocrine disorders, epilepsy and migraine, malignant disorders, thrombosis or disorders of the female genital tract: E00E3, G40G43, C00C97, I80, I82, I26 and/or N70N98). Both crude and adjusted values shown. Adjustments were made for age, family income, educational level and health care utilization.
(XLSX)

S4 Table AUC change. **Change in area under the receiver operating curve (AUC) with addition of hormonal contraceptive use to a model containing individual predictors**. Age stratified change in the area under the curve for use of psychotropic drugs when adding information on use of hormonal contraceptives (ATC G02BA, G02BB, G03AA, G03AB, G03AC) to a model already including age, family income, highest educational level in family, previous hospitalizations, outpatient hospital visits and having a diagnosis of thromboembolism, epilepsy or migraine, or menstrual disturbances including endometriosis in the 815 662 Swedish women.
(DOCX)
