# Supplementary material for: Hormonal contraception increases the risk of psychotropic drug use in adolescent girls but not in adults: A pharmacoepidemiological study on 800 000 Swedish women
Source: PLoS One. 2018 Mar 22;13(3):e0194773. doi: 10.1371/journal.pone.0194773 (PMC5864056; doi:10.1371/journal.pone.0194773)
Supplement: S1 Table — Prevalence of different hormonal contraceptive methods in 2010–2011 by contraceptive type in our cohort of 815 662 Swedish women aged 12–39. All combined HC contain estrogen in addition to progesterone, except for G03AA14 (Zoely), which contains estradiol. (DOCX) [file pone.0194773.s001.docx]

| **S1 Table.** | | | | | | | | |
| --- | --- | --- | --- | --- | --- | --- | --- | --- |
|  | | **ATC code** | | | **Type** | **Progesterone** | *n* | % |
| ***Oral methods*** | | | | |  |  |  |  |
|  | **Combined HC** | |  | |  |  |  |  |
|  | G03AA03* | | COC | | Lynestrenol | 258 | 0.1 |  |
|  | G03AA05 | | COC | | Norethisterone | 2416 | 0.6 |  |
|  | G03AA07 | | COC | | Levonorgestrel | 130 468 | 31.7 |  |
|  | G03AA09 | | COC | | Desogestrel | 7378 | 1.8 |  |
|  | G03AA11 | | COC | | Norgestimate | 24 260 | 5.9 |  |
|  | G03AA12 | | COC | | Drospirenon | 55 755 | 13.5 |  |
|  | G03AA14* | | COC | | Nomegestrol | 43 | 0.0 |  |
|  | G03AB | | COC | | ** | 1773 | 0.4 |  |
|  | G03AB03 | | COC | | Levonorgestrel | 19 167 | 4.7 |  |
|  | G03AB04 | | COC | | Norethisterone | 9511 | 2.3 |  |
|  | G03AB05* | | COC | | Desogestrel | 460 | 0.1 |  |
|  | *All COC users* | | | |  | *251 489* | *60.1* |  |
|  | **Progesterone-only HC** | | | |  |  |  |  |
|  | G03AC01 | | POP | | Norethisterone | 5517 | 1.3 |  |
|  | G03AC02 | | POP | | Lynestrenol | 4301 | 1.0 |  |
|  | G03AC09 | | POP | | Desogestrel | 94 617 | 23 |  |
|  | *All POP users* | | | |  | *104 435* | *25.4* |  |
| ***Non-oral methods*** | | |  | |  |  |  |  |
|  | **Combined HC** | |  | |  |  |  |  |
|  | G02BB01 | | Ring | | Etonogestrel | 22 950 | 5.6 |  |
|  | G03AA13 | | Patch | | Norelgestromin | 4507 | 1.1 |  |
|  | *All ring/patch users* | | | | | *27 457* | *6.7* |  |
|  | **Progesterone-only HC** | | | |  |  |  |  |
|  | G02BA03 | | IUD | | Levonorgestrel | 8956 | 2.2 |  |
|  | G03AC03* | | Implant | | Levonorgestrel | 67 | 0,0 |  |
|  | G03AC06 | | Injection | | Medroxiprogesteron | 3223 | 0.8 |  |
|  | G03AC08 | | Implant | | Etonogestrel | 15 932 | 3.9 |  |
|  | | *All IUD/implant/injection users* | | | | | *28 178* | *6.8* |
|  | | |  |  | |  |  |  |
| *Total HC users* | | | |  | |  | *411 559* | *100* |
| HC; Hormonal contraceptive, COC; combined oral contraceptives, POP; progesterone-only pills, Patch; skin patch (Evra), Ring; intravaginal ring (NuvaRing), IUD; Intrauterine device.  *ATC codes with few users due to upcoming deregistration at the time for analysis, with the exception of G03AA14, which instead was newly registered.  **Unclassifiable because of incomplete information on the ATC code. | | | | | | | | |
