# Supplementary material for: Hormonal contraception increases the risk of psychotropic drug use in adolescent girls but not in adults: A pharmacoepidemiological study on 800 000 Swedish women
Source: PLoS One. 2018 Mar 22;13(3):e0194773. doi: 10.1371/journal.pone.0194773 (PMC5864056; doi:10.1371/journal.pone.0194773)
Supplement: S2 Table — Odds ratios (OR) with 95% confidence intervals (CI) and area under the curve (AUC), stratified on adolescents and adults, for the association between use of different hormonal contraceptives and a first time use of psychotropic drugs within a one-year follow-up after baseline (2010–2011) in 815 662 Swedish women. (DOCX) [file pone.0194773.s002.docx]

| **S2 Table.** | | | | |
| --- | --- | --- | --- | --- |
| ***Age 12–19 years*** | |  |  |  |
|  |  |  | Crude model | Adjusted model ^a^ |
| **ATC code** | **HC type** | **Progesterone** | **OR (95% CI)** | **OR (95% CI)** |
| Non-user |  |  | 1.00 (ref) | 1.00 (ref) |
| G03AA05 | COC | Norethisterone | 1.80 (1.07–3.00) | 1.19 (0.71–2.00) |
| G03AA07 | COC | Levonorgestrel | 1.94 (1.84–2.06) | 1.51 (1.42–1.60) |
| G03AA09 | COC | Desogestrel | 2.51 (1.82–3.48) | 1.61 (1.16–2.24) |
| G03AA11 | COC | Norgestimate | 2.33 (2.07–2.62) | 1.69 (1.50–1.91) |
| G03AA12 | COC | Drospirenon | 2.92 (2.69–3.17) | 2.05 (1.87–2.24) |
| G03AB03 | COC | Levonorgestrel | 1.67 (1.32–2.11) | 1.19 (0.94–1.51) |
| G03AB04 | COC | Norethisterone | 2.19 (1.72–2.81) | 1.54 (1.20–1.97) |
| G03AC01 | POP | Norethisterone | 2.07 (1.31–3.27) | 1.25 (0.79–1.98) |
| G03AC02 | POP | Lynestrenol | 3.77 (2.52–5.65) | 2.23 (1.47–3.38) |
| G03AC09 | POP | Desogestrel | 2.72 (2.54–2.91) | 1.93 (1.80–2.08) |
| G02BB01 | Ring | Etonogestrel | 3.01 (2.60–3.49) | 2.05 (1.77–2.39) |
| G03AA13 | Patch | Norelgestromin | 3.69 (2.96–4.60) | 2.46 (1.97–3.07) |
| G02BA03 | IUD | Levonorgestrel | 5.26 (4.05–6.83) | 2.90 (2.22–3.79) |
| G03AC06 | Injection | Medroxyprogesterone | 4.33 (2.69–6.99) | 2.37 (1.46–3.84) |
| G03AC08 | Implant | Etonogestrel | 3.55 (3.15–4.00) | 2.36 (2.08–2.67) |
| *AUC* |  |  | *0.62 (0.61–0.63)* | *0.68 (0.67–0.68)* |
| ***Age 20–30 years*** | |  |  |  |
|  |  |  | Crude model | Adjusted model ^a^ |
| **ATC code** | **HC type** | **Progesterone** | **OR (95% CI)** | **OR (95% CI)** |
| Non user |  |  | 1.00 (ref) | 1.00 (ref) |
| G03AA05 | COC | Norethisterone | 0.72 (0.54–0.95) | 0.79 (0.60–1.05) |
| G03AA07 | COC | Levonorgestrel | 0.83 (0.79–0.88) | 0.89 (0.85–0.94) |
| G03AA09 | COC | Desogestrel | 0.80 (0.69–0.92) | 0.84 (0.72–0.97) |
| G03AA11 | COC | Norgestimate | 0.87 (0.79–0.95) | 0.95 (0.87–1.05) |
| G03AA12 | COC | Drospirenon | 1.23 (1.17–1.30) | 1.30 (1.23–1.37) |
| G03AB03 | COC | Levonorgestrel | 0.77 (0.70–0.85) | 0.82 (0.75–0.91) |
| G03AB04 | COC | Norethisterone | 0.83 (0.72–0.94) | 0.88 (0.77–1.00) |
| G03AC01 | POP | Norethisterone | 0.99 (0.85–1.15) | 0.96 (0.82–1.11) |
| G03AC02 | POP | Lynestrenol | 0.99 (0.84–1.17) | 0.96 (0.81–1.14) |
| G03AC09 | POP | Desogestrel | 1.01 (0.97–1.06) | 1.01 (0.96–1.05) |
| G02BB01 | Ring | Etonogestrel | 1.25 (1.16–1.34) | 1.31 (1.22–1.41) |
| G03AA13 | Patch | Norelgestromin | 1.26 (1.05–1.50) | 1.20 (1.01–1.43) |
| G02BA03 | IUD | Levonorgestrel | 1.23 (1.11–1.37) | 1.08 (0.97–1.20) |
| G03AC06 | Injection | Medroxyprogesterone | 1.92 (1.67–2.22) | 1.56 (1.34–1.82) |
| G03AC08 | Implant | Etonogestrel | 1.18 (1.07–1.30) | 1.14 (1.03–1.26) |
| *AUC* |  |  | *0.54 (0.53–0.54)* | *0.60 (0.60–0.60)* |
| HC; Hormonal contraceptive, COC; combined oral contraceptives, POP; progesterone-only pills, Patch; skin patch (Evra), Ring; intravaginal ring (NuvaRing), IUD; Intrauterine device.  ^a^Adjusted for age, family income, highest educational level in family, previous hospitalizations, outpatient hospital visits, and having a diagnosis of thromboembolism, epilepsy or migraine, or menstrual disturbances including endometriosis.  *1773 cases were excluded due to having only a five-digit ATC code available  **The following ATC codes were excluded due to few users, because of upcoming deregistration or being newly registered at the time of analysis G03AA03 (n=285), G03AB05 (n=480), G03AC03 (n=67)) | | | | |
