# Supplementary material for: Hormonal contraception increases the risk of psychotropic drug use in adolescent girls but not in adults: A pharmacoepidemiological study on 800 000 Swedish women
Source: PLoS One. 2018 Mar 22;13(3):e0194773. doi: 10.1371/journal.pone.0194773 (PMC5864056; doi:10.1371/journal.pone.0194773)
Supplement: S4 Table — Change in area under the receiver operating curve (AUC) with addition of hormonal contraceptive use to a model containing individual predictors. Age stratified change in the area under the curve for use of psychotropic drugs when adding information on use of hormonal contraceptives (ATC G02BA, G02BB, G03AA, G03AB, G03AC) to a model already including age, family income, highest educational level in family, previous hospitalizations, outpatient hospital visits and having a diagnosis of thromboembolism, epilepsy or migraine, or menstrual disturbances including endometriosis in the 815 662 Swedish women. (DOCX) [file pone.0194773.s004.docx]

**S2 Table**

| Age |  | AUC (95% CI) | AUC change |
| --- | --- | --- | --- |
|  |  |  |  |
| 12–14 | Without HC | 0.65 (0.64 - 0.67) |  |
|  | Adding HC | 0.69 (0.67 - 0.70) | 0.04 |
|  |  |  |  |
| 15–17 | Without HC | 0.61 (0.60 - 0.61) |  |
|  | Adding HC | 0.63 (0.62 - 0.64) | 0.02 |
|  |  |  |  |
| 18–20 | Without HC | 0.60 (0.59 - 0.61) |  |
|  | Adding HC | 0.60 (0.60 - 0.61) | 0.00 |
|  |  |  |  |
| 21–25 | Without HC | 0.59 (0.58 - 0.60) |  |
|  | Adding HC | 0.59 (0.58 - 0.60) | 0.00 |
|  |  |  |  |
| 26–30 | Without HC | 0.59 (0.58 - 0.59) |  |
|  | Adding HC | 0.59 (0.58 - 0.59) | 0.00 |
|  |  |  |  |

AUC; Area under the curve, HC; Hormonal contraception
